# Novel metasurface phase-modulation mechanism

**DOI:** 10.1038/s41377-021-00629-z

**Published:** 2021-09-14

**Authors:** Qinghua Song

**Affiliations:** Tsinghua Shenzhen International Graduate School, Tsinghua University, Shenzhen, 518000 China

**Keywords:** Nanophotonics and plasmonics, Metamaterials

*Science, 373(6559), 1133–1137, (2021)*

10.1126/science.abj3179

The past few years have witnessed exciting developments in non-Hermitian physics, showing unconventional phenomena and unique features associated with exceptional points (EPs). EPs exist in many open systems, leading to a spectral singularity. The research team from CNRS-CRHEA in France collaborating with the University of California, Berkeley in US utilizes the topological feature around an EP to introduce a novel design in metasurface to achieve a new wavefront phase encoding technique. They show that the intriguing polarization response of singular plasmonic meta-atoms encircling an EP leads to 2π-phase modulation on a chosen outgoing channel, which is topologically protected by the EP. In auxiliary, combining the exceptional topological phase with Pancharatnam-Berry phase, they achieve arbitrary wavefront engineering on cross polarization channels independently. Their breakthrough explorations not only provide a new degree of freedom to address optical phase in a full 2π range, but also open the way to a new class of optical and photonic applications.

